# Case Report: Stevens–Johnson syndrome/toxic epidermal necrolysis overlap with ocular and urogenital involvement in an extremely preterm infant following broad-spectrum antibiotic exposure

**DOI:** 10.3389/fmed.2026.1854901

**Published:** 2026-06-22

**Authors:** Adnan Hadid, Lana A. Shaiba, Mona Alkallabi, Khalid Nabil Nagshabandi, Nora Al-Saud, Rodhan Khaled Alnahdi, Layan Alsanad, Maee Barakeh, Naif Ahmed Alshehri, Khalid Faisal Alkahtani, Adel Abdulaziz Alsuhaibani, Abdulmalik Abdulaziz Alharbi

**Affiliations:** 1 Department of Pediatric, College of Medicine, King Saud University, Riyadh, Saudi Arabia; 2 Department of Neonatology, King Saud University Medical City, Riyadh, Saudi Arabia; 3 Department of Dermatology, College of Medicine, King Saud University, Riyadh, Saudi Arabia; 4 Department of Pediatric, King Saud University Medical City, Riyadh, Saudi Arabia

**Keywords:** drug hypersensitivity, extreme prematurity, necrotizing enterocolitis, neonatal sepsis, Stevens–Johnson syndrome, toxic epidermal necrolysis

## Abstract

Stevens–Johnson syndrome/toxic epidermal necrolysis (SJS/TEN) is a rare, life-threatening epidermolytic reaction characterized by widespread keratinocyte necrosis, epidermal detachment, and mucosal involvement. In neonates, particularly extremely premature infants, diagnosis is challenging because several mimickers are more common in the neonatal intensive care unit (NICU), and evidence guiding systemic therapy is limited. We report a male infant born at 23 + 5 weeks’ gestation (birth weight 620 g) who developed progressive mucocutaneous erosions and flaccid bullae at a corrected gestational age of 35 + 3 weeks (day of life [DOL] 83, weight 1800 g). The eruption began periorally and spread to the trunk, extremities, and genitals, with ocular and urethral involvement. Epidermal detachment was estimated at 25–30% body surface area (BSA), consistent with SJS/TEN overlap. Skin biopsy demonstrated widespread full-thickness keratinocyte necrosis with sub-epidermal blistering and sparse lymphocytic inflammation; direct immunofluorescence was negative. Following protocolized supportive care and adjunctive intravenous immunoglobulin (IVIG), cutaneous progression halted with re-epithelialization. The infant later died from septic shock with multi-organ failure related to extreme prematurity and recurrent necrotizing enterocolitis (NEC). This case highlights the diagnostic complexity of epidermolytic syndromes in extremely premature infants and supports early suspicion, prompt withdrawal of potential triggers, and protocolized multidisciplinary supportive care.

## 1 Introduction

Stevens–Johnson syndrome (SJS) and toxic epidermal necrolysis (TEN) represent a spectrum of life-threatening epidermolytic drug reactions characterized by extensive keratinocyte death, painful erythema, blistering with skin detachment, and prominent mucosal involvement. These entities are differentiated by the extent of epidermal detachment: SJS (<10% BSA), SJS/TEN overlap (10–30%), and TEN (>30%) (1). The underlying pathobiology involves immune-mediated cytotoxic processes culminating in widespread epidermal necrosis; medications are the predominant triggers in most cohorts, while infections and other exposures may also contribute (2, 3).

SJS/TEN in neonates is exceptionally rare. The estimated overall incidence ranges from 1 to 6 cases per million person-years, and the incidence in neonates is thought to be significantly lower (4, 5). Recent pediatric cohorts report that antimicrobials and anti-epileptics dominate as triggers, that ocular involvement occurs in the majority of cases, and that mortality remains substantial despite supportive care (6). However, these series are populated almost exclusively by older infants and children, and extremely premature neonates are essentially absent from the published evidence base. The few neonatal reports available are isolated case descriptions, leaving clinicians without a structured framework for diagnosis or treatment in this uniquely vulnerable subgroup, and no consensus management framework currently exists. Extremely premature infants are routinely exposed to numerous pharmacological agents during prolonged NICU stays, yet drug hypersensitivity reactions are infrequently reported, potentially owing to the immaturity of the neonatal immune system or under recognition.

Diagnosis is challenging because mimickers, such as staphylococcal scalded skin syndrome (SSSS), infectious blistering disorders, autoimmune bullous disease, nutritional or deficiency-related dermatoses, and inherited epidermolysis bullosa, are more common in this age group. Clinicopathologic correlation is essential; biopsy often demonstrates full-thickness epidermal necrosis with sub-epidermal blistering, while negative direct immunofluorescence helps exclude autoimmune blistering disease (2). Management priorities include prompt withdrawal of suspected culprit drugs, meticulous supportive care, and early multidisciplinary involvement—particularly ophthalmology given the frequency and long-term impact of ocular disease (2, 7). Systemic immunomodulatory therapies remain debated, with evidence largely derived from non-neonatal populations (2, 8).

Here, we report an extremely premature infant who developed an SJS/TEN overlap phenotype with multimucosal involvement temporally associated with broad-spectrum antimicrobial exposure. Our case directly addresses the gap in the literature by providing biopsy-confirmed clinicopathological data, a detailed temporal drug-association analysis, and a protocolized management approach in an infant born at 23 + 5 weeks’ gestation, which is among the youngest gestational ages reported with SJS/TEN overlap. This case highlights the diagnostic complexity of epidermolytic syndromes in the NICU and the practical considerations in management of this uniquely vulnerable population.

## 2 Case description

### 2.1 Patient background

A male infant was born at 23 weeks and 5 days of gestation via spontaneous vaginal delivery with a birth weight of 620 g and was admitted to the NICU immediately for management of extreme prematurity. The neonatal course was complicated by respiratory distress syndrome requiring prolonged mechanical ventilation, bronchopulmonary dysplasia, recurrent necrotizing enterocolitis (NEC) including perforating enterocolitis, and recurrent late-onset sepsis with multiple organisms (*Enterococcus faecalis*, *Staphylococcus haemolyticus*, and *Klebsiella pneumoniae*). He required prolonged total parenteral nutrition (TPN) and seven sequential courses of broad-spectrum antimicrobials (Table 1). A right encysted hydrocele of the spermatic cord was identified on scrotal ultrasound. No family history of blistering or autoimmune skin disease was reported.

**Table 1:** Clinical timeline illustrating the temporal relationship between antimicrobial exposure, laboratory findings, cutaneous eruption, and key clinical events.

DOL	CGA	Antimicrobial events	Clinical events	Key lab findings
1	23 + 6 wk	Course 1: ampicillin, gentamicin, cefotaxime	Birth (620 g); RDS; NICU admission	WBC 13.63; Plt 260; Hb 11.8
4	24 + 2 wk	Fluconazole prophylaxis started	—	—
10	25 + 1 wk	Course 2: Tazocin → Amp/Gent → Vanc/Gent → Vanc (10 d)	Omphalitis/sepsis	—
26	27 + 3 wk	Course 3: Vanc + Amikacin + Ampho B → Tazocin + Vanc (7 d)	VAP	—
32	28 + 2 wk	Fluconazole stopped	—	—
42	29 + 5 wk	Course 4: Meropenem + Amikacin + Vanc (14 d)	Sepsis/NEC Stage II	—
72	33 + 0 wk	Course 5: Vanc + Amikacin + Metronidazole (14 d)	Recurrent NEC	—
80	34 + 1 wk	Course 5 completed	—	WBC 7.8; Plt 171; ANC 1.61; AST 14
83	35 + 3 wk	—	Rash onset: perioral erythema and erosions (1800 g)	No labs obtained
88	35 + 5 wk	Course 6: Vanc + Tazocin started/stopped	Dermatology consult; SJS/TEN dx; suspect drugs stopped; IVIG started	WBC 3.8; Plt 145; ANC 0.62; CRP 34.3
90	36 + 0 wk	—	Skin biopsy obtained	—
91–92	36 + 1–36 + 2 wk	—	Re-epithelialization begins; no new lesions	—
94	36 + 3 wk	Course 7: Meropenem + Linezolid + Amikacin + Ampho B	NEC recurrence; exploratory laparotomy	—
Later	—	—	Septic shock; multiorgan failure; death	Polymicrobial BSI
Summary	83–~92	—	~8-9 days from rash onset to halting of cutaneous progression and onset of re-epithelialization	—

CGA, corrected gestational age; DOL, day of life; ANC, absolute neutrophil count; Plt, platelets; BSI, bloodstream infection; MOF, multiorgan failure.

The mother was a 35-year-old woman, gravida 2, para 1, with bronchial asthma and a prior cesarean section, admitted for preterm labor. She completed antenatal dexamethasone. Screening for Group B Streptococcus and herpes simplex virus (HSV) was negative. She did not receive antenatal or postnatal antibiotics or antiepileptic medications. No maternal history of drug allergy was reported.

### 2.2 Onset and progression of the cutaneous eruption

At a corrected gestational age of approximately 35 weeks and 3 days (DOL 83, weight approximately 1800 g), the patient developed new mucocutaneous findings three days after completion of the fifth antibiotic course (vancomycin, amikacin, and metronidazole) during an otherwise clinically stable period. No additional medications had been introduced immediately preceding onset.

The eruption initially manifested as perioral erythema and erosions, progressing over 8–10 days to hemorrhagic crusting and flaccid bullae. The initial impression was adhesive-related or pressure injury, and topical mupirocin was commenced. Over 72 h, lesions spread to the genital area, trunk, and extremities in a centrifugal pattern.

**Figure 1:**
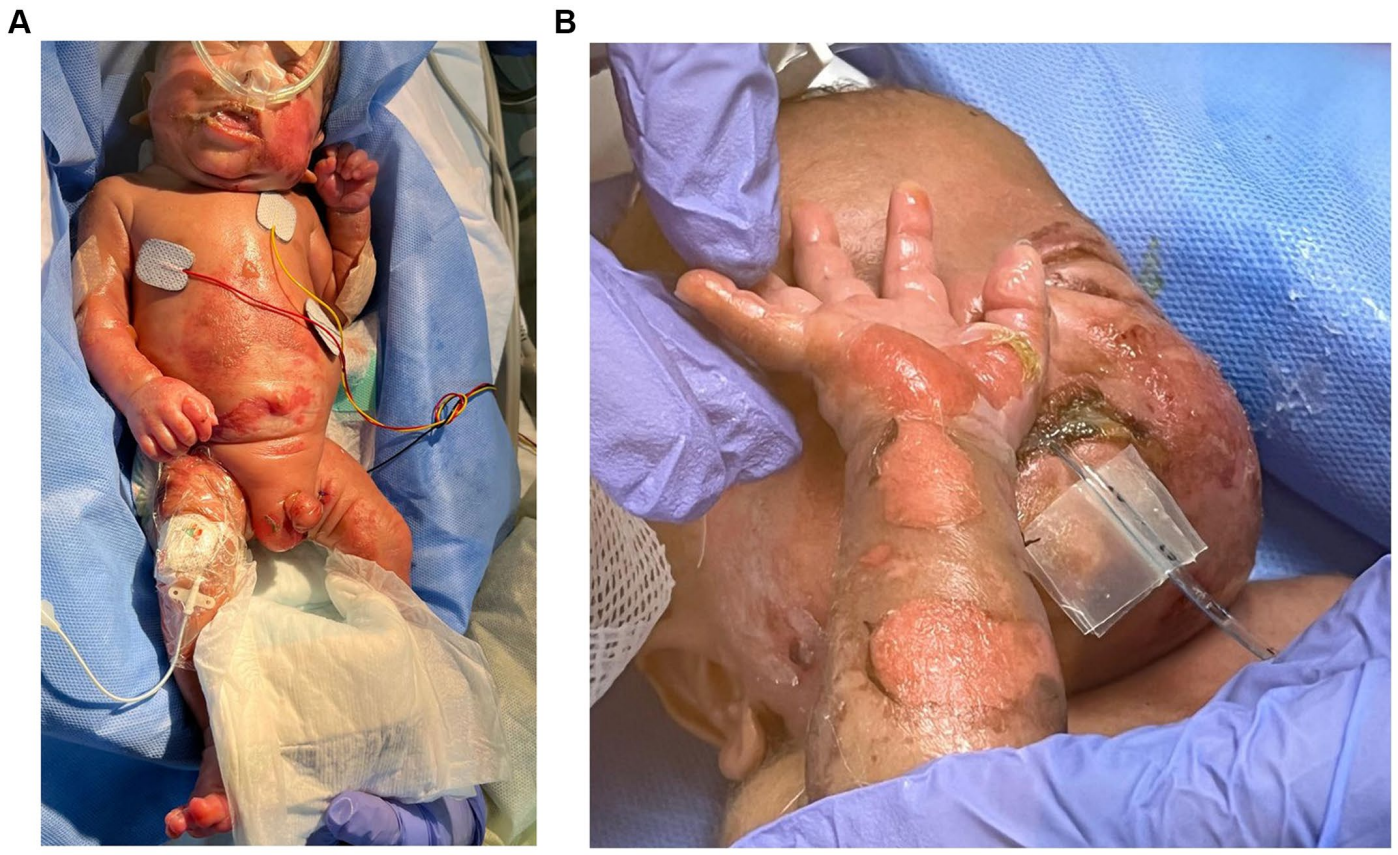
Multiple well-demarcated dusky erythematous patches and plaques with blistering, erosions, and epidermal detachment, involving the face, trunk, extremities, and genital region. **(A)** Dusky erythematous patches with epidermal detachment over the trunk (white arrows). **(B)** Perioral hemorrhagic crusting (black arrows).

On formal dermatological re-evaluation, examination revealed multiple well-demarcated dusky erythematous patches and plaques with erosions, blisters, and epidermal detachment (Figure 1). The perioral region showed crusted erosions with hemorrhagic crusting. The upper extremities had eroded blisters with intact grouped vesicles on an erythematous base, particularly bilateral elbows and palms. The trunk showed dusky erythematous plaques that evolved into erosions and blisters with epidermal detachment. The lower extremities had grouped vesicles over the left thigh and a bulla on the right thigh. The genital region showed penile and pubic edema with urethral mucosal involvement. Estimated epidermal detachment was 25–30% BSA, consistent with SJS/TEN overlap. The Nikolsky sign was negative. Hair and nail examinations were normal with no milia or scars.

### 2.3 Mucosal involvement

Oral mucosal involvement was evident but difficult to fully assess due to ongoing nasogastric tube support. Ophthalmologic examination was performed at the bedside by a pediatric ophthalmologist using a portable slit lamp; fluorescein staining was not feasible in this extremely premature, ventilated infant due to clinical instability and risk–benefit considerations. Ocular involvement included marked eyelid edema with epithelial erosion and desquamation; the palpebral conjunctiva was hyperemic with a papillary reaction and minimal membrane formation with mucoid discharge. No symblepharon or conjunctival adhesions were identified; the ophthalmologic impression was SJS with left eye involvement. Urologic assessment was performed by a pediatric urologist, with documentation of meatal, glans, and pubic involvement; urethral mucosal erosions with associated genital edema were also present.

### 2.4 Histopathological findings

**Figure 2:**
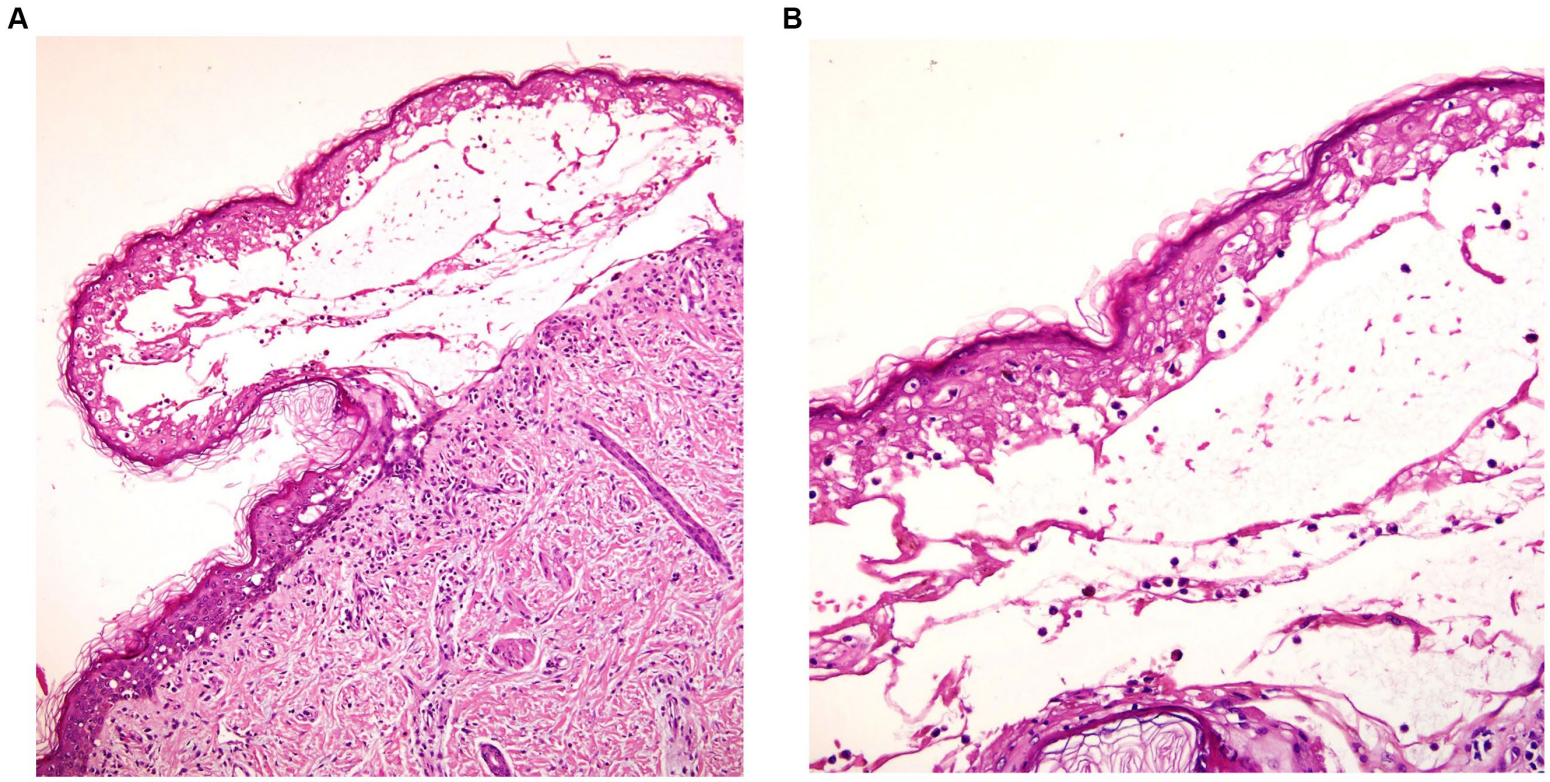
**(A)** Extensive full-thickness epidermal necrosis with sub-epidermal separation resulting in blister formation (H&E stain, ×100), with arrow indicating sub-epidermal cleft. **(B)** High-power view demonstrates necrotic keratinocytes, marked epidermal cell death, and clefting at the dermo-epidermal junction, with minimal inflammatory infiltrate (H&E stain, ×400), with arrowheads indicating dermo-epidermal junction clefting.

Two 3-mm punch biopsies were obtained on DOL 90 (Figure 2): one from a fresh blister edge for hematoxylin and eosin (H&E) staining and one from clinically uninvolved perilesional skin (within 1 cm of an active lesion) for direct immunofluorescence (DIF). The DIF specimen was transported in Michel’s medium. Histopathology revealed widespread full-thickness epidermal necrosis with complete epidermal–dermal separation, consistent with a subepidermal blistering process. Numerous necrotic keratinocytes and marked destruction of the epidermal architecture were observed, with prominent dermo-epidermal junction clefting and only sparse dermal inflammatory infiltrate. These findings are characteristic of SJS/TEN (2, 9, 10). DIF was negative for IgG, IgM, IgA, C3, and fibrinogen, arguing against autoimmune bullous dermatoses (2).

### 2.5 Differential diagnosis and suspected causative agents

The differential diagnosis included SSSS (excluded by negative cultures and full-thickness necrosis on biopsy), bullous impetigo (negative wound cultures), HSV infection (negative HSV-1/2 IgM and PCR), cutaneous candidiasis (no histological or culture evidence), autoimmune blistering disease (negative DIF), epidermolysis bullosa (less likely given subacute onset, temporal drug relation, negative Nikolsky sign, absent milia/scars, and improvement after drug withdrawal), and acrodermatitis enteropathica (clinical pattern and histology more consistent with drug reaction). The clinicopathologic findings collectively favored SJS/TEN overlap.

The eruption occurred in the context of sequential antibiotic exposure, limiting identification of a single culprit drug. The temporal association with the fifth course (vancomycin, amikacin, and metronidazole) raised suspicion for one or more of these agents. Vancomycin has been rarely implicated in SJS/TEN, and metronidazole has been infrequently associated with SJS. Clinical improvement after drug discontinuation supports a drug-related etiology. The Algorithm of Drug Causality for Epidermal Necrolysis (ALDEN) was not applied prospectively, given the retrospective nature of the assessment and the overlapping sequential antimicrobial exposure that precluded identification of a single culprit drug.

### 2.6 Laboratory findings

Notable laboratory trends between pre-rash (DOL 80) and during-rash (DOL 88) time points included progressive leukopenia (WBC from 7.8 to 3.8 × 10^9^/L) with absolute neutropenia (from 1.61 to 0.62 × 10^9^/L), thrombocytopenia (platelets from 171 to 145 × 10^9^/L), a rise in AST (from 14 to 53.9 U/L), conjugated hyperbilirubinemia (direct bilirubin from 14 to 49 μmol/L), and elevated inflammatory markers (CRP 34.3 mg/L; procalcitonin 0.17 ng/mL). HSV-1/2 IgM was negative.

### 2.7 Management

All non-essential and potentially causative medications were discontinued on DOL 88. The patient was managed with a protocolized neonatal SJS/TEN approach, itemized below by type, rationale, dose/route/frequency/duration, modifications during the course, and concurrent measures, in accordance with CARE guideline items 9 and 10:

- Thermoregulation and minimal handling: Maintained in an incubator with controlled humidity to support epidermal barrier function; avoidance of adhesives.
- Wound care: Non-adherent dressings with sterile petrolatum gauze; gentle cleansing with warm saline; topical antimicrobials applied to eroded areas. Topical mupirocin was discontinued at the time of dermatology consultation.
- Fluid and electrolyte management: Strict monitoring with tailored intravenous fluids titrated to insensible losses through denuded skin.
- Nutrition: Continuation of parenteral nutrition with gradual reintroduction of enteral feeds as tolerated.
- Ocular care: Topical antibiotic ointments, preservative-free lubricating drops every 2 h, and corticosteroid drops per ophthalmology; consultation initiated at diagnosis.
- Urologic mucosal care: Careful hygiene of meatal and genital regions, per pediatric urology.
- Analgesia: Opioid analgesia titrated to comfort, with avoidance of nonsteroidal anti-inflammatory drugs to limit nephrotoxicity and platelet inhibition.
- Adjuvant immunomodulation: IVIG 1 g/kg/dose intravenously daily for four consecutive days, considered on the basis of disease severity, biologic plausibility, and an acceptable safety profile. No IVIG-attributable adverse effects were observed.
- Concurrent measures: Infection-control reinforcement and multidisciplinary consultation (dermatology, ophthalmology, urology, infectious diseases, and wound care). Empiric antimicrobial coverage was subsequently adjusted following isolation of *Stenotrophomonas maltophilia*.

### 2.8 Treatment response

Within days of initiating therapy, no new lesions developed, and existing erosions began to re-epithelialize (Figure 2B). Mucosal lesions improved progressively. Repeat wound cultures isolated scant multidrug-resistant *Stenotrophomonas maltophilia*, prompting infection control reinforcement without systemic sepsis. Follow-up confirmed steady epithelial recovery and mucosal inflammation resolution.

### 2.9 Clinical timeline

Table 1 presents the chronological timeline illustrating the temporal relationship between antimicrobial exposure, laboratory investigations, the cutaneous eruption, and key clinical events.

### 2.10 Subsequent clinical course

On DOL 94, the infant developed a third episode of NEC with colonic distension. A contrast study demonstrated a stricture at the splenic flexure. Exploratory laparotomy revealed two colonic strictures with perforation; segmental resection with primary anastomosis was performed. The postoperative course was complicated by polymicrobial wound and bloodstream infections (*Klebsiella pneumoniae*, *Candida albicans*, *Candida parapsilosis*, *Pseudomonas fluorescens*, and *Stenotrophomonas maltophilia*). The infant subsequently deteriorated with septic shock and multiorgan failure in the setting of extreme prematurity and recurrent intra-abdominal disease, leading to death. Long-term dermatological and ophthalmological sequelae could not be evaluated.

### 2.11 Key findings

In summary, the principal findings of this case were (i) biopsy-confirmed full-thickness keratinocyte necrosis with negative DIF; (ii) 25–30% BSA epidermal detachment consistent with SJS/TEN overlap; (iii) multimucosal involvement (oral, ocular, and urogenital); (iv) temporal association with the fifth antimicrobial course (vancomycin, amikacin, and metronidazole); and (v) halting of cutaneous progression and re-epithelialization following drug withdrawal and IVIG.

## 3 Discussion

The diagnosis of SJS/TEN in premature neonates is frequently delayed due to clinical overlap with more common neonatal blistering disorders, including bullous impetigo, inherited epidermolysis bullosa, nutritional dermatoses, SSSS, and other infectious or metabolic conditions (2, 9, 11). In this case, the initial impression of adhesive-related injury delayed consideration of a drug reaction. The morphological progression from erythematous patches to grouped vesicles, bullae, and crusted erosions—combined with perioral predilection and multifocal distribution—ultimately prompted re-evaluation. The diagnosis was supported by extensive epidermal detachment, multi-mucosal involvement, and histopathology demonstrating full-thickness keratinocyte necrosis with negative DIF (2, 9, 10). Published data in neonates are limited to isolated case reports and small pediatric series, so most therapeutic principles are extrapolated from older populations (2, 6, 9, 10). Of note, the classical Nikolsky sign may be unreliable in extremely premature, fragile skin, and the implication is that morphology combined with biopsy carries greater diagnostic weight than bedside signs alone.

Several mechanistic considerations are relevant to why classical drug-induced SJS/TEN is uncommon in extremely preterm infants. The relative immaturity of T-cell–mediated cytotoxicity and the incomplete adaptive immune repertoire at <28 weeks would, *a priori*, be expected to dampen the immune-mediated keratinocyte killing characteristic of SJS/TEN. The occurrence of a full-blown SJS/TEN overlap phenotype in our patient therefore raises the plausibility of an alternative or threshold mechanism, including cumulative drug exposure across seven sequential antimicrobial courses, septic and inflammatory priming, and possible HLA-restricted susceptibility (acknowledging that HLA typing was not performed).

Ocular and urogenital involvement are particularly important determinants of long-term morbidity in pediatric SJS/TEN. Acute ocular involvement has been reported in the majority of affected children and, without timely intervention, chronic sequelae such as symblepharon, corneal scarring, and visual loss are common (6, 11). Genitourinary involvement ranges from dysuria and meatal erosions to adhesions and strictures, and early urologic input is recommended (6, 12). In this infant, early ophthalmologic and urologic care with intensive lubrication and topical therapy was followed by clinical improvement prior to deterioration from unrelated causes.

The laboratory data demonstrated progressive leukopenia with absolute neutropenia, thrombocytopenia, rising AST, and conjugated hyper-bilirubinemia, potentially reflecting drug-induced marrow suppression, the inflammatory process of SJS/TEN, hepatic involvement, or the metabolic burden of critical illness. The mildly elevated inflammatory markers with negative blood cultures argued against fulminant sepsis as the primary driver of the eruption, further supporting a drug-induced etiology.

IVIG has been proposed to interfere with Fas-mediated keratinocyte apoptosis; however, evidence in neonates is limited to individual case reports and small case series, and adult study results have been inconsistent (2, 6, 13). Within our limited inferential capacity, we cannot disentangle the relative contributions of culprit-drug withdrawal, intensive supportive care, and IVIG to the observed cutaneous stabilization. All three were initiated within a narrow temporal window, and prompt withdrawal of the offending drug is, on its own, the single intervention most consistently associated with improved outcomes in SJS/TEN. The biologic rationale for IVIG rests on *in vitro* data suggesting that pooled human immunoglobulin can interfere with Fas/FasL-mediated keratinocyte apoptosis. Clinical evidence in adults remains heterogeneous, with some studies and meta-analyses suggesting a mortality benefit at higher cumulative doses (≥3 g/kg) and others showing no significant effect. Evidence in neonates is restricted to isolated case reports and small series, none of which are controlled, and direct extrapolation from adult data is problematic given developmental differences in immune function, drug metabolism, and skin barrier physiology in extremely premature infants. We therefore present IVIG in this case as an adjunctive therapy considered on the basis of disease severity, biologic plausibility, and an acceptable safety profile rather than as a treatment of demonstrated benefit. Nevertheless, IVIG is often considered in severe presentations because of its comparatively acceptable safety profile. In this case, clinical progression halted after IVIG alongside intensive supportive care. Although causality cannot be established, IVIG may represent a reasonable adjunctive option in carefully selected severe neonatal cases (6, 13).

Several factors influence outcome in extremely premature infants with SJS/TEN. First, the extent of epidermal detachment is a key prognostic variable, although established adult prognostic instruments such as SCORTEN have not been validated in neonates and may underestimate risk in this population. Second, time-to-recognition and time-to-drug-withdrawal are the most consistently modifiable determinants of outcome across age groups. Third, infection control is central given baseline NICU colonization with multidrug-resistant organisms and the loss of skin-barrier function. Fourth, prematurity-related comorbidities (NEC, BPD, and late-onset sepsis) exert a disproportionate impact and in our case ultimately drove the fatal outcome despite cutaneous recovery. Finally, early multidisciplinary involvement (dermatology, ophthalmology, urology, infectious diseases, surgery, and wound care) is essential.

Practical recommendations for suspected SJS/TEN in extremely premature infants:

(1) Maintain a high index of suspicion when progressive blistering develops following antimicrobial or antiepileptic exposure; do not anchor on adhesive injury or SSSS without re-evaluation.
(2) Obtain dermatology consultation and a skin biopsy (lesional H&E plus perilesional DIF) within 24–48 h of suspected onset.
(3) Discontinue all non-essential medications; for essential drugs, substitute with structurally unrelated agents where feasible. Apply ALDEN scoring prospectively to guide drug attribution.
(4) Estimate body surface area involvement carefully and reassess daily; epidermal detachment may progress for 5–7 days.
(5) Manage in a thermoneutral, high-humidity environment with non-adherent dressings, sterile petrolatum gauze, and minimal handling; avoid all adhesive products.
(6) Initiate ophthalmology and urology consultation at diagnosis, regardless of apparent severity, given the impact of mucosal sequelae on long-term outcome.
(7) Reinforce infection-control measures and obtain baseline surveillance cultures; anticipate colonization with multidrug-resistant organisms and select empiric antimicrobials accordingly should secondary infection occur.
(8) Consider IVIG (1 g/kg/day for 3-4 days) as adjunctive therapy in severe cases after multidisciplinary discussion, recognizing the limited evidence base in neonates.
(9) Document the case prospectively with photographs, BSA estimates, treatment timing, and laboratory trends to support future pooled analyses.

To anchor our case within the wider experience of neonatal and pediatric SJS/TEN, Table 2 compares the present case with previously reported neonatal and early-infant cases and with a recent pediatric cohort. Three observations emerge from this comparison: (1) the present case is, to our knowledge, among the most premature reported, with most prior neonatal cases occurring in term infants; (2) antimicrobials are the most frequently implicated trigger across the published neonatal experience, with antiepileptics being the next most common in term and preterm cases; and (3) survival in the published neonatal series is generally favorable when prompt drug withdrawal and supportive care are instituted, with mortality more often related to underlying comorbidities than to the cutaneous disease itself, as observed in our patient.

**Table 2:** Selected reported neonatal/early-infant SJS/TEN cases compared with the present case.

Case	GA/age at onset	Suspected trigger(s)	Treatment	Complications	Outcome
Present case (2026)	23 + 5 wk; DOL 83 (CGA 35 + 3)	Vancomycin, amikacin, metronidazole (sequential courses)	Drug withdrawal, supportive care, IVIG 1 g/kg × 4 d, ophthalmologic & urologic care	Ocular & urethral involvement; recurrent NEC; polymicrobial sepsis	Skin recovery; later death from septic shock/MOF (NEC-related)
Fernandes et al. (10)	Term newborn (DOL 7)	Reported antibiotic exposure	Supportive care; corticosteroids	Mucosal involvement	Survived
Khalaf et al. (14)	Preterm (≈32 wk); neonatal period	Phenobarbital	Drug withdrawal, supportive care, IVIG	Mucosal involvement	Survived
Hsu et al. (15)	Term neonate	Co-trimoxazole/antibiotics	Supportive care; IVIG	Ocular involvement	Survived
Scully and Frieden (16)	Neonate (≤4 wk)	Drug-induced (multiple agents)	Supportive care	Multi-mucosal	Variable
Rahman et al. (6) cohort	Pediatric cohort (incl. infants)	Antibiotics, anti-epileptics	Supportive care; IVIG in severe cases; corticosteroids	Ocular sequelae most common	Mortality 8–12% in cohort

GA, gestational age; DOL, day of life; CGA, corrected gestational age; NEC, necrotizing enterocolitis; MOF, multiorgan failure; IVIG, intravenous immunoglobulin.

Three concrete implications for NICU practice emerge from this case: (1) maintaining a low threshold for dermatology consultation when atypical mucocutaneous lesions develop after antimicrobial exposure; (2) the value of early biopsy with paired H&E and DIF to differentiate SJS/TEN from SSSS, autoimmune blistering disease, and epidermolysis bullosa; and (3) the importance of structured, multidisciplinary supportive care (dermatology, ophthalmology, urology, infectious diseases, wound care, and nutrition).

This premature patient demonstrated early disease stabilization with halted progression and gradual re-epithelialization, though the infant later died from causes related to extreme prematurity and recurrent intra-abdominal disease. This emphasizes that prompt recognition and protocolized management may limit disease progression, while overall prognosis may remain strongly influenced by underlying comorbidities (6, 10).

### 3.1 Limitations

Several limitations warrant acknowledgment. As a single-patient report, our findings cannot be generalized to all extremely premature infants and are subject to selection and reporting bias. The case is described retrospectively, raising the possibility of recall bias regarding the precise sequence of clinical events. The overlapping antimicrobial exposure precluded identification of a single causative agent; the ALDEN was not applied prospectively; genetic testing for epidermolysis bullosa was deferred; and long-term outcomes could not be assessed. Co-occurring sepsis and recurrent NEC may also have contributed to the inflammatory and laboratory abnormalities observed. HLA typing was not performed. Bedside ophthalmologic assessment was constrained by the infant’s clinical instability and ventilatory support, precluding fluorescein staining and detailed slit-lamp documentation, and no serial photographic documentation of the ocular surface with fluorescein was obtained at presentation or follow-up. Long-term dermatological, ophthalmological, urological, and neurodevelopmental outcomes could not be assessed because the infant died from causes unrelated to SJS/TEN before such evaluation was feasible. Hematological, hepatic, and inflammatory laboratory derangements may reflect contributions from extreme prematurity, recurrent sepsis, and TPN-related cholestasis in addition to SJS/TEN itself, limiting the specificity of these findings. Finally, our supportive-care protocol reflects local NICU practice and may not be directly transferable to centers with different resource availability.

## 4 Patient perspective

The patient’s parents were informed about the diagnosis and management throughout the clinical course. The family expressed understanding of the complexity of the condition in the context of extreme prematurity and provided written informed consent for publication of this case report and accompanying images.

## 5 Conclusion

This report contributes to the limited literature on SJS/TEN overlap in extremely premature infants. It highlights the need for heightened vigilance when neonates present with progressive blistering and mucosal involvement following medication exposure. Early dermatological consultation and skin biopsy are essential when atypical eruptions develop. A structured, multidisciplinary approach involving dermatology, ophthalmology, urology, infectious diseases, genetics, neonatology, and wound care teams is critical for optimal management. Continued reporting of neonatal cases is necessary to better characterize triggers, refine management approaches, and inform adjunctive therapy use in this vulnerable population.

## Data Availability

The original contributions presented in the study are included in the article/supplementary material, further inquiries can be directed to the corresponding author.

## Author’s note

Stevens–Johnson syndrome/toxic epidermal necrolysis overlap should be considered when extremely premature neonates develop progressive mucocutaneous blistering following antimicrobial exposure. Early dermatological consultation, skin biopsy, and protocolized multidisciplinary supportive care are essential to limit disease progression in this vulnerable population.
